# CARING FOR HEALTH AND CULTURE: INSIGHTS INTO DISEASE MANAGEMENT, CAREGIVING, AND WELL-BEING IN BLACK COMMUNITIES

**DOI:** 10.1093/geroni/igae098.0453

**Published:** 2024-12-31

**Authors:** Darlingtina Esiaka, Manka Nkimbeng

**Affiliations:** Chair; Co-Chair

## Abstract

There has been growing advocacy for a nuanced understanding of health practices and challenges in diverse Black contexts in recent years. This symposium aims to show the multifaceted intersection of health, culture, and well-being within Black communities, offering a comprehensive exploration of chronic disease management, family caregiving, and mental well-being. Ado-Mensah scrutinizes the nuanced roles and characteristics of family caregivers in African American and Black immigrant communities, shedding light on the unique challenges they face in the context of diabetes management. Groves introduces a novel approach to hypertension management by combining the DASH diet with culturally tailored recipes, recognizing the importance of culturally sensitive interventions for improving health outcomes among Black women. Kum explores the cultural aspects influencing family caregiving practices among African and Afro-Caribbean immigrants, contributing valuable insights into the broader context of eldercare within these communities. Lastly, Hutton sheds light on mental health challenges, emphasizing the need for a nuanced understanding of depression and loneliness symptoms within the diverse diasporic experiences of African Americans and Black communities. Together, these contributions weave a rich tapestry of knowledge, providing valuable insights into disease management, caregiving dynamics, and mental well-being in the intricate intersection of health and culture within Black communities.

